# The Views of Hospital Secretaries

**Published:** 1911-05-27

**Authors:** 


### THE VIEWS OF HOSPITAL SECRETARIES.

**Mr. HARRY JOHNSON, House Governor of the Leicester Infirmary, writes:—**

The probable effect of the National Insurance Bill on the Leicester Saturday Hospital Society (as typical of a multitude of like bodies) has been detailed in some points just made by Sir Edward Wood, the Chairman of the Board of the Infirmary and the Founder and President of the Society. No one is better qualified to express an opinion. It may be of interest to note that he spoke most hopefully of the future of the society, and while of opinion that the contributions of the working classes had now reached their zenith (£13,800 per annum) he did not think that, when the working of the new Act was well established, there would be a serious diminution of income of the society, although he was prepared for a small falling-off at first. His opinion was governed by the following considerations:

1. Membership of the Saturday Hospital Society was covered by the payment of 1d. per week. In addition to the goodwill of the working classes to the infirmary, which they had largely helped to render efficient, their support carried with it substantial representation on the Board and Committees. The society already maintained a convalescent home for men of forty-eight beds, which benefited some 700 men every year. A second home for female convalescents was now approaching completion.

2. Members of the society were entitled to three weeks' free treatment in the respective homes on the recommendation of their medical advisers. Pending the opening of the new home, female patients were being accommodated in other institutions at the expense of the society.

3. Male members enjoyed the privilege of securing for their wives free convalescent benefits. Sir Edward Wood was of opinion that these among other privileges were the society's surest hope for a continuation of contributions. The members were the beneficial owners and occupiers of the homes, and were conferring inestimable benefits upon their fellow workers, and he, personally, had little doubt that the beneficent work of the society would be maintained.

4. It should not be forgotten that not a small proportion of the members were already members of male or female friendly societies. Their sickness contributions under the Bill would be greater, and would, in effect, be transferred from one account to another, and there was no reason to anticipate their withdrawal from membership.

He thought, however, that the Bill would render some of the work of the out-patients' department superfluous, and the Board would probably insist on it being used for consultative purposes by insured persons. This in itself would provide relief to the existing congestion, and would enable eligible non-insured persons to be more efficiently treated.

**Mr. RICHARD J. COLE, Secretary-Superintendent of Addenbrooke's Hospital, Cambridge, writes:—**

So far as I have been able to peruse the National Insurance Bill, and taking a general view of the matter, I am of opinion that in the event of the said Bill becoming law the work of provincial general hospitals will be decreased very little, if at all; in fact, I should not be surprised if the number of patients both in and out were to increase, as certain factors which exist now and are responsible for a large number of patients presenting themselves at our hospitals, will not be removed by the enactment of this Bill.

Although the Bill provides for the wage-earners, apparently it does not do so for the wives and children; therefore it may be fairly assumed that these will seek treatment at our general hospitals.

I am further of opinion that the Bill will tend to diminish the income of voluntary hospitals—to what extent it is difficult to predict at present. I shall give the Bill careful consideration, and write again in due course.

